# Preoperative prediction of WHO/ISUP grade of ccRCC using intratumoral and peritumoral habitat imaging: multicenter study

**DOI:** 10.1186/s40644-025-00875-z

**Published:** 2025-05-03

**Authors:** Zhihui Chen, Hongqing Zhu, Hongmin Shu, Jianbo Zhang, Kangchen Gu, Wenjun Yao

**Affiliations:** 1 Department of Radiology, The Second Affiliated Hospital of Anhui Medical University, Hefei, Anhui China; 2 Medical Imaging Research Center, Anhui Medical University, Hefei, Anhui China; 3 Department of Radiology, The First Affiliated Hospital of Anhui Medical University, Hefei, Anhui China

**Keywords:** Clear cell renal cell carcinoma, Computed tomography, Habitat analysis, Radiomics, WHO/ISUP grading

## Abstract

**Objectives:**

The World Health Organization/International Society of Urological Pathology (WHO/ISUP) grading of clear cell renal cell carcinoma (ccRCC) is crucial for prognosis and treatment planning. This study aims to predict the grade using intratumoral and peritumoral subregional CT radiomics analysis for better clinical interventions.

**Methods:**

Data from two hospitals included 513 ccRCC patients, who were divided into training (70%), validation (30%), and an external validation set (testing) of 67 patients. Using ITK-SNAP, two radiologists annotated tumor regions of interest (ROI) and extended surrounding areas by 1 mm, 3 mm, and 5 mm. The K-means clustering algorithm divided the tumor region into three sub-regions, and the Least Absolute Shrinkage and Selection Operator (LASSO) regression identified the most predictive features. Various machine learning models were established, including radiomics models, peritumoral radiomics models, models based on intratumoral heterogeneity (ITH) score, clinical models, and comprehensive models. Predictive ability was evaluated using receiver operating characteristic (ROC) curves, area under the curve (AUC) values, DeLong tests, calibration curves, and decision curves.

**Results:**

The combined model showed strong predictive power with an AUC of 0.852 (95% CI: 0.725–0.979) on the test data, outperforming individual models. The ITH score model was highly precise, with AUCs of 0.891 (95% CI: 0.854–0.927) in training, 0.877 (95% CI: 0.814–0.941) in validation, and 0.847 (95% CI: 0.725–0.969) in testing, proving its superior predictive ability across datasets.

**Conclusion:**

A comprehensive model combining Habitat, Peri1mm, and salient clinical features was significantly more accurate in predicting ccRCC pathologic grading.

**Key points:**

**Question**: Characterize tumor heterogeneity to non-invasively predict WHO/ISUP pathological grading preoperatively.

**Findings**: An integrated model combining subregion characterization, peritumoral characteristics, and clinical features can predict ccRCC grade preoperatively.

**Clinical relevance**: Subregion tumor characterization outperforms the single-entity approach. The integrated model, compared with the radiomics model, boosts grading and prognostic accuracy for more targeted clinical actions.

**Supplementary Information:**

The online version contains supplementary material available at 10.1186/s40644-025-00875-z.

## Critical relevance statement

The non-invasive preoperative pathological grading methods for ccRCC are still imperfect. This study significantly improves the prediction accuracy of ccRCC pathological grading by integrating intratumoral and peritumoral habitat imaging with significant clinical features.

## Introduction

Renal cell carcinoma (RCC) is the most frequently encountered malignant neoplasm of the urinary tract, with clear cell renal cell carcinoma (ccRCC) being the predominant histological subtype, accounting for 70–90% of all RCC diagnoses [1]. ccRCC is notable for its elevated propensity for metastasis and a less favorable prognosis compared to other RCC subtypes [2, 3], thereby significantly impacting patient survival outcomes. Therapeutic approaches for ccRCC encompass a range of interventions, including surgical interventions (radical nephrectomy and nephron-sparing surgery), radiotherapy, chemotherapy, immunotherapy, and targeted therapy [4–6]. The diverse nature of ccRCC contributes to marked heterogeneity in patient outcomes. Empirical studies [7, 8] suggest that histopathological grading is a crucial independent prognostic indicator, affecting both tumor recurrence and patient survival. The World Health Organization/International Society of Urological Pathology (WHO/ISUP) grading system, as developed by the World Health Organization (WHO) in 2016, is the standard grading system in clinical use, stratifying tumors into four distinct grades, with advancing grades associated with worsening clinical outcomes [9, 10]. Nevertheless, the determination of pathological grade is conventionally performed on surgical specimens, and preoperative assessment relies on invasive procedures such as needle biopsy, which are fraught with limitations including procedural invasiveness and the risk of sampling error, potentially leading to misclassification due to tumor heterogeneity [11]. Therefore, the identification of non-invasive techniques for the preoperative evaluation of the WHO/ISUP grade in ccRCC is of paramount importance for accurately assessing tumor biology, informing therapeutic decision-making, and prognosticating patient outcomes.

Among the various preoperative screening modalities for renal cancer, computed tomography (CT) is the most extensively utilized. Nonetheless, CT alone is not yet capable of predicting pathologic grading or providing comprehensive prognostic assessments. Radiomics, as a non-invasive research approach, offers the ability to delve into tumor heterogeneity by extracting a wealth of features from medical images, thereby increasingly demonstrating its advantage in the diagnosis and pathologic grading of ccRCC [12, 13]. However, conventional radiomics typically treats the tumor as a monolithic entity, overlooking the regional phenotypic diversity within the tumor [14]. Intratumoral heterogeneity pertains to the genetic and phenotypic discrepancies among distinct tumor regions. These disparities result from repeated cell division and proliferation during tumor development, which alter the molecular biology or genetic profile of tumor subclones [15]. ccRCC, being a tumor with high heterogeneity, encompasses varied components such as necrosis, cystic degeneration, and hemorrhage. These distinct regions may exhibit different biological behaviors, which can influence the tumor's growth, response to treatment, and ultimate prognosis [16].

**Fig. 1:**
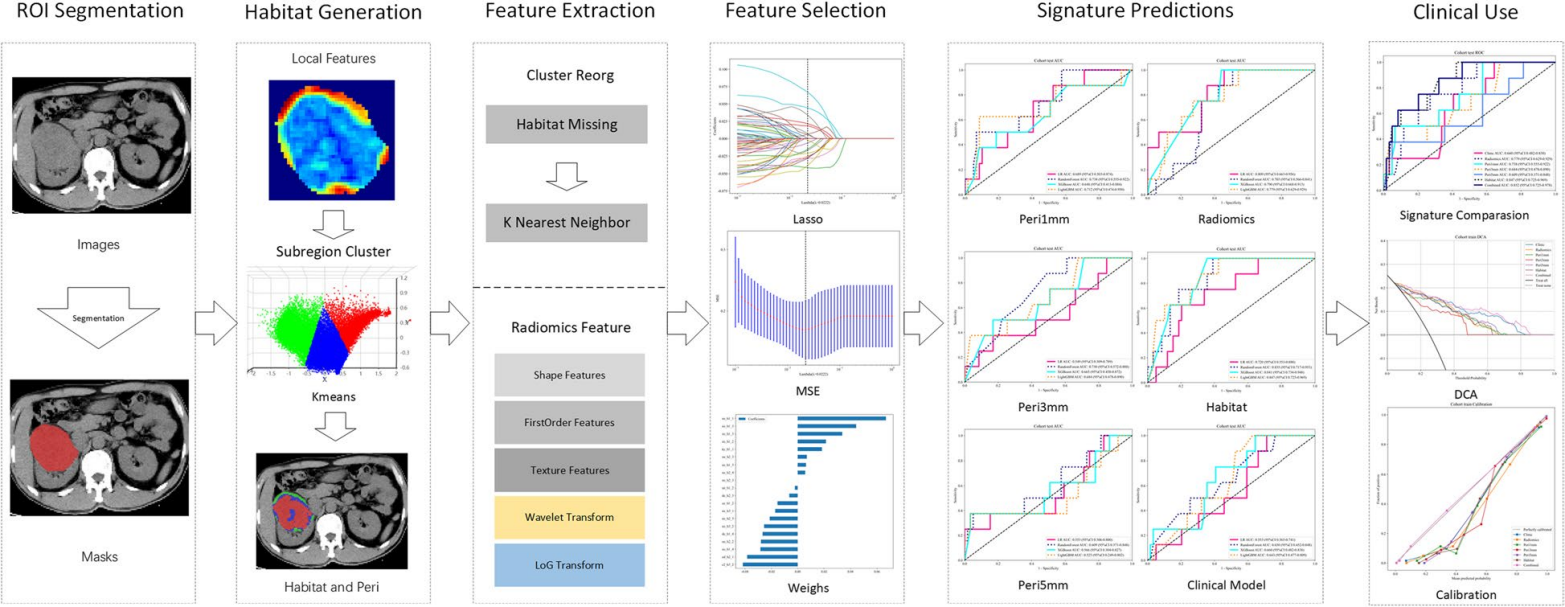
Overall workflow of this study. ROI: regions of interest; LASSO: Least Absolute Shrinkage and Selection Operator; MSE: Mean Standard Error; Peri: Peritumoral; DCA: Decision Curves Analysis

In pursuit of advancing personalized treatment strategies, the advent of habitat imaging has introduced a groundbreaking approach. This radiomics-based technique involves the detailed extraction and systematic analysis of imaging features from medical imaging datasets, with the aim of characterizing and quantifying the intrinsic heterogeneity of tissues, as well as elucidating the interactions among distinct tumor compartments [17]. The primary intent of habitat imaging is to stratify the tumor into various subregions, each exhibiting homogeneous characteristics [18], thereby facilitating a more nuanced understanding of tumor heterogeneity [19]. Previous studies have focused on the relationship between intra-tumoral heterogeneity and clinical outcomes [20, 21]. However, the application of this concept to tumor grading has been less extensively investigated. Moreover, the majority of these studies have predominantly employed magnetic resonance imaging (MRI) as the imaging modality [14, 22], with few studies in the application of habitat analysis to renal cancer using CT scans—despite CT being the preferred imaging technique for the clinical evaluation of ccRCC. Therefore, our study aims to identify subregions with similar characteristics from multiphase contrast-enhanced CT images, evaluating the predictive utility of a radiomics model that incorporates the heterogeneity features of these subregions for the preoperative pathological grading of ccRCC patients. Furthermore, we propose to synthesize a comprehensive model by amalgamating habitat imaging with the peritumoral attributes of ccRCC patients, thereby assessing the predictive strength of an integrated dataset on the pathological grading of ccRCC. Figure 1 provides an illustrative outline of our methodological workflow.

## Methods

### Patients

We retrospectively collected CT imaging data and related clinical information of patients diagnosed with ccRCC at the Second Affiliated Hospital of Anhui Medical University in China from August 2012 to August 2023. This study was approved by the Ethics Committee (No. YX2024-219). Since this study is a retrospective analysis, informed consent was waived. The inclusion criteria for this study were: pathologically confirmed ccRCC; complete preoperative CT examination; no previous surgery or other interventional treatment. The exclusion criteria were: incomplete clinical data; CT images that could not be further analyzed; presence of malignancies in other organs. Patients were grouped according to the postoperative pathological WHO/ISUP results, with grades I-II classified as the low-grade group and grades III-IV as the high-grade group. A total of 446 patients were included, comprising 294 males and 152 females. These patients were divided into two groups: a training cohort (70%) and a validation cohort (30%). Additionally, to assess the generalizability of the model, we included data from 67 ccRCC patients from the First Affiliated Hospital of Anhui Medical University in China as an external validation set. The gold standard adopted in this study was the histopathological diagnosis of the tissue after surgical resection. All the included cases were pathologically confirmed as ccRCC after the operation, and then were graded by pathologists according to the WHO/ISUP grading criteria.

### Image acquisition

CT scans with Siemens SOMATOM Force, Definition AS40 and Philips Brilliance iCT. Patients were positioned in the supine position and instructed to hold their breath during the scan. The scanning range extended from the xiphoid process to the anterior superior iliac spine. The scan parameters were as follows: 120 kV, adaptive current, 5 mm slice (thickness and interval), 512 × 512 matrix. The protocol included an initial non-contrast abdominal scan, followed by contrast administration. A total of 80 mL of iodixanol (300 mgI/mL) was administered intravenously into the median cubital vein at a rate of 2.5 mL/s using an injector. Subsequent scans were performed during the cortical (with a delay of 25–30 s), parenchymal (60–70 s), and secretory (120–150 s) phases, respectively, and covered the same range as the initial non-contrast scan.

### Image segmentation

Two experienced radiologists (HQ. Z and ZH. C), without prior knowledge of the pathological conditions, independently used ITK-SNAP software to manually delineate the entire tumor region layer by layer to obtain the region of interest (ROI). The delineation range does not include perirenal and renal sinus fat. In instances where divergences in delineation occurred, a radiologist (WJ. Y) with twenty years of experience resolved these discrepancies, ensuring the accuracy and reliability of the regions of interest (ROI) identification process.

### Data preprocessing

In our research work, we implemented a fixed-resolution resampling method to standardize the voxel intervals for all analyzed samples. At the same time, we set the display range of Hounsfield units (HUs). Specifically, the window width was set to 300 and the window position was set to 25. This standardized processing of data was done to ensure accurate comparative analysis between images.

### Intratumor heterogeneity analysis

We used four unique imaging sequences during our study: the non-contrast phase, the renal cortical phase, the renal parenchymal phase, and the excretory phase. These sequences were analyzed independently, with each phase employing specific clustering analysis and workflows for constructing peritumoral regions for our study.

### Peritumoral region dilation

**Fig. 2:**
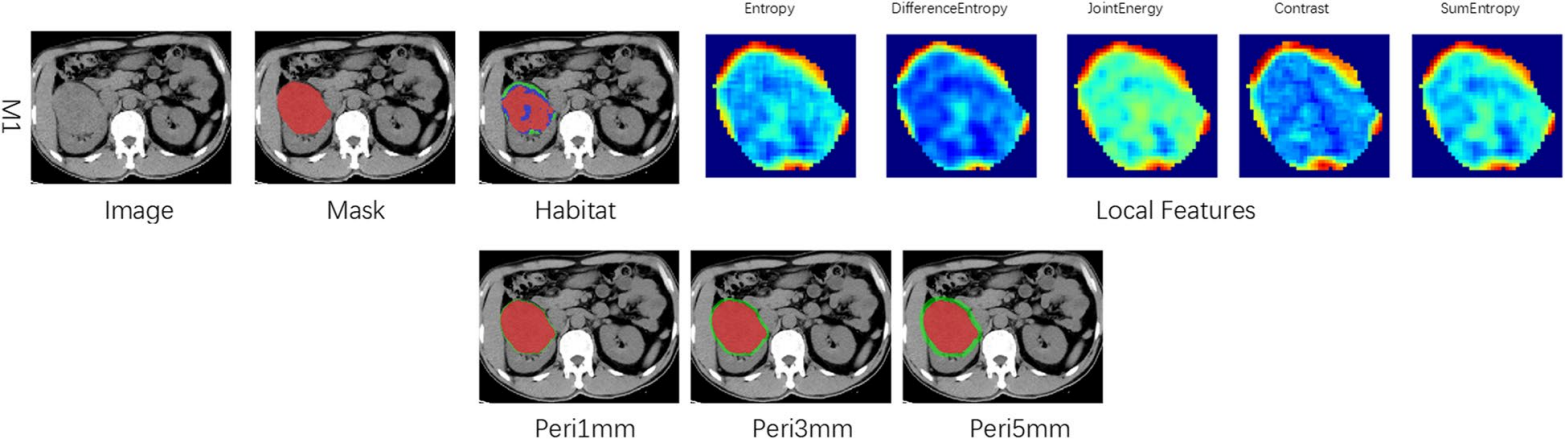
Presents the Peritumoral and Intratumor Heterogeneity regions generated. Red indicates the intra-tumoral region, and green represents the peritumoral region. The peritumoral region was expanded at intervals of 1 mm, 3 mm, and 5 mm

The peritumoral area surrounding the tumor is of significant importance for specific medical research. Using a platform’s mask filling toolkit, we systematically expanded the original ROI mask by incrementally increasing the radial distance to evaluate the impact of these expanded areas on the predictive performance of our model. We set expansion intervals of 1 mm, 3 mm, and 5 mm to investigate the effects of different extents of the peritumoral region on predictive accuracy. Figure 2 illustrates these expanded peritumoral regions, vividly depicting the step-by-step expansion process.

### Subregion generation

In this study, we extracted local features like entropy and energy from each voxel in the VOI via CT images. We used a 5 × 5 × 5 moving window method to calculate them, getting a 19-dimensional feature vector per voxel, as detailed in Supplementary 1A. Then, we applied the K-means clustering algorithm to divide the VOI into several discrete subregions (with 3 to 10 clusters). To optimize the segmentation, we used the Calinski-Harabasz (CH) score to find the best number of clusters. This helped us detail the tumor's heterogeneity and understand its structural complexity better.

### Feature extraction

Handcrafted radiomics features are grouped into geometric (tumor shape/size), intensity (voxel brightness), and texture (GLCM, GLRLM, GLSZM, and NGTDM etc. for spatial patterns). Analyzed VOI and tumor subregions. KNN managed clustering gaps for label consistency. Extracted features per IBSI with pyradiomics 3.0.1. Pre-fusion combined subregion features for better tumor grading prediction.

### Feature selection

We used the Intraclass Correlation Coefficient (ICC) to evaluate the consistency of ROI delineation. In the experiment, we retained features with ICC > 0.8 to reduce the impact of delineation on the features.

For relevance analysis, first used Pearson's correlation coefficient to eliminate highly correlated features (0.9 threshold). Then, mRMR method reduced feature set to 64 by balancing correlation and independence. Finally, obtained radiomic signature via Least Absolute Shrinkage and Selection Operator (LASSO) regression. LASSO penalizes regression coefficients to filter out unnecessary features. Optimal regularization parameter λ determined by ten-fold cross-validation to keep only the most predictive features in the model. The steps from initial screening to LASSO-based model simplification ensure radiomics markers are predictive and stable.

### Signature building

In our research work, we constructed a series of machine learning models designed to predict Radiomics Signature, Peritumoral Signature, and ITH score, which utilize features screened by the LASSO technique. We optimized the parameters of the models through a five-fold cross-validation and Grid-Search algorithm. In addition, during the training process, the Synthetic Minority Over-sampling Technique (SMOTE) was employed to sample the training set, to solve the class imbalance problem. At the same time, as an essential metric for evaluating the predictive performance of a model, the Area Under the Receiver Operating Characteristic Curve is independent of the proportion of positive and negative samples, making it particularly suitable for addressing scenarios involving class imbalance.

Radiomics Signature: we used logistic regression for linear modeling and combined algorithms such as Random Forest, XGBoost, and LightGBM to deal with more complex data structures, and constructed a risk assessment model that accurately captures the details of the data through a selected set of features.

Peri-tumor radiomics Signature (PeriXmm): The “X” here refers to the peri-tumor region. We combine intra- and peri-tumor features, applying the same feature screening and machine learning algorithms as for the intra-tumor radiomics signature.

Intra-Tumor Heterogeneity Score (ITH Score): Our clustering algorithm has an unsupervised feature, which allows us to avoid Intraclass Correlation Coefficient (ICC) analysis, thus ensuring model stability and effectively assessing intra-tumor heterogeneity.

Clinical Signature: we screened clinical features with *p*-values less than 0.05 by univariate and multivariate analyses, which were used to construct clinical prediction models.

Integrated model: we further validated our integrated model by univariate and stepwise multivariate analyses that included only clinical features with *p*-values less than 0.05 and combined them with the best Peritumoral Signature and Habitat Signature to form the final integrated model. This approach ensured a comprehensive assessment and integration of clinical predictors, thereby improving the predictive accuracy of the model.

### Statistical analysis

We applied the Shapiro–Wilk test to check the clinical data's normal distribution. For continuous variables, we chose the t-test or Mann–Whitney U-test based on data distribution to assess statistical significance. *P*-values over 0.05 between groups indicated no significant difference and verified fair grouping. All statistical analyses were done on the OnekeyAI platform (version 3.5.12) in Python 3.7.12. We used Statsmodels (version 0.13.2) for calculations, PyRadiomics (version 3.0.1) for extracting radiomics features, and Scikit-learn (version 1.0.2) for implementing machine learning algorithms like Support Vector Machines (SVM).

## Results

This study included an aggregate of 513 patients with ccRCC, among which 403 patients were classified as low-grade according to WHO/ISUP criteria, and 110 patients were classified as high-grade.

### Clinical features

We collected clinical data, including demographic information, clinical manifestations, laboratory tests, tumor imaging and morphology (Supplementary Table), to identify clinical features associated with tumor grading and to construct a clinical prediction model. After univariate and multivariate analyses, the characteristics of gender, age, and platelets showed significant differences with *p*-values below 0.05 in the statistical analysis (Table 1). Gender differences can affect tumor biological behavior and treatment response. Age is a key prognostic factor, and higher age may mean a poorer prognosis. Platelet count changes may relate to tumor aggressiveness and the patient’s coagulation function. Based on these findings, we’ll include these indicators as clinical control factors in later studies to analyze their impact further.

**Table 1:** Univariable and multivariable analysis of clinical features

Feature_name	OR	OR lower95%CI	OR upper95%CI	*p*_value	OR	OR lower95%CI	OR upper95%CI	*p*_value
Sex(female/male)	1. 113	1. 022	1. 212	< 0. 05	1. 132	1. 034	1. 24	< 0. 05
Age(mean ± SD)	1. 004	1. 001	1. 008	< 0. 05	1. 005	1. 001	1. 008	< 0. 05
Hematuria (absent/present)	1. 311	1. 14	1. 508	< 0. 05	1. 161	1. 011	1. 332	0. 075
Abdominalpain (absent/present)	1. 071	0. 969	1. 184	0. 258				
Abdominalmass (absent/present)	0. 947	0. 685	1. 31	0. 784				
Tumorhistory (absent/present)	1. 207	1. 026	1. 419	0. 056				
Location (left/right)	1. 039	0. 958	1. 127	0. 435				
eGFR (ml/(min·1. 73 m2))	0. 998	0. 995	1. 001	0. 188				
BUN(mmol/L)	1. 025	1. 001	1. 049	0. 085				
Cr(umol/L)	1	0. 999	1. 001	0. 773				
CHO(mmol/L)	0. 957	0. 908	1. 009	0. 169				
ALB(g/L)	0. 988	0. 979	0. 996	< 0. 05	0. 998	0. 989	1. 007	0. 683
Hb(g/L)	0. 996	0. 994	0. 999	< 0. 05	0. 999	0. 996	1. 002	0. 6
PLT(g/L)	1. 001	1. 001	1. 002	< 0. 05	1. 001	1	1. 002	< 0. 05
NE(10 × 10^9)	1. 037	1. 012	1. 063	< 0. 05	1. 001	0. 975	1. 026	0. 974
LYM(10 × 10^9)	1. 021	0. 989	1. 055	0. 285				
MO(10 × 10^9)	1. 185	0. 956	1. 471	0. 194				
R(mm)	1. 004	1. 002	1. 006	< 0. 05	1. 001	0. 999	1. 003	0. 621
E	0. 961	0. 896	1. 031	0. 358				
N	1. 099	1. 042	1. 16	< 0. 05	1. 067	0. 992	1. 147	0. 142
A	1. 064	1. 013	1. 116	< 0. 05	1. 009	0. 963	1. 059	0. 745
L	1. 071	1. 02	1. 125	< 0. 05	0. 995	0. 931	1. 063	0. 895
H(no/yes)	1. 376	1. 239	1. 528	< 0. 05	1. 168	1. 022	1. 335	0. 056
Renal_score	1. 051	1. 028	1. 074	< 0. 05	1. 005	0. 966	1. 046	0. 848
Map1	1. 002	0. 944	1. 063	0. 965				
Map2	1. 047	1. 01	1. 084	< 0. 05	0. 984	0. 946	1. 022	0. 488
Map_score	1. 027	0. 998	1. 055	0. 128				
Cystic (absent/present)	0. 963	0. 868	1. 068	0. 552				
Capsule (absent/present)	1. 014	0. 929	1. 108	0. 79				
MVD(absent/present)	1. 016	0. 932	1. 106	0. 765				
Calcification (absent/present)	1. 127	1. 015	1. 251	0. 061				
^Invasion(no/yes)^	1. 39	1. 247	1. 548	< 0. 05	1. 097	0. 96	1. 255	0. 254
Cavainvolvement (absent/present)	1. 373	1. 165	1. 618	< 0. 05	1. 032	0. 85	1. 254	0. 79
Lymphnodes (absent/present)	1. 459	1. 194	1. 784	< 0. 05	1. 054	0. 831	1. 338	0. 715
Metastasis(absent/present)	2. 136	1. 556	2. 933	< 0. 05	1. 277	0. 894	1. 824	0. 259
Enhancement1 (heterogeneous/homogeneous)	1. 121	0. 972	1. 292	0. 186				
Enhancement2(absent/present)	0. 924	0. 847	1. 007	0. 132				

R = maximum tumor diameter, E = exophytic/endophytic, N = distance between the tumor and the renal sinus and collecting system, A = tumor located on the ventral or dorsal side of the kidney, L = tumor location along the longitudinal axis of the kidney, H = whether the tumor invades the renal pedicle vessels, Map1 = posterior perirenal fat thickness at the level of the renal vein, Map2 = perirenal fat involvement, MVD = intratumoral blood vessels, Enhancement2 = fast in and fast out

### Habitat generation and feature extraction

**Fig. 3:**
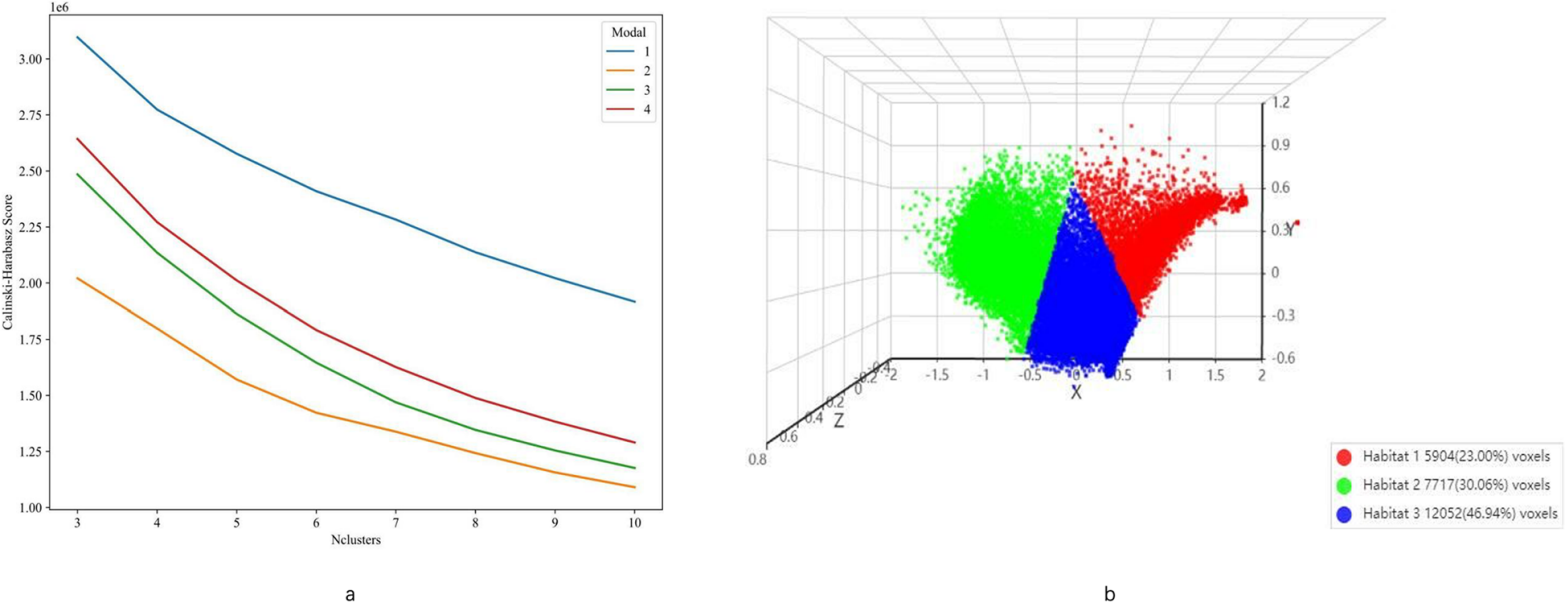
**a** Presents the Calinski-Harabasz (CH) scores for different numbers of clusters, illustrating the impact of the number of clusters on the segmentation effect for each pattern. **b** provides a visualization of the DCE features, segmented into three distinct clusters. DCE: Dynamic Contrast-Enhanced

We evaluated the impact of clustering centers (3 to 10) on results. Based on the highest CH index (Fig. 3a), 3 was the optimal number as all sequences had the highest CH index with it (Fig. 3b). 1,106 unique radiomic features were extracted and grouped into shape, first-order, and texture types (216 first-order, 14 shape, others texture). The final ITH score combines features from 3 sub-regions (13,272 features in total). Radiomics and Peri-Tumor features each have 4,424 features. Feature extraction was performed using a custom Pyradiomics tool. See its documentation for details. Our study has a chart showing feature category distribution for an overview of their proportions (Supplementary Fig. 1).

### The results of feature selection

**Fig. 4:**
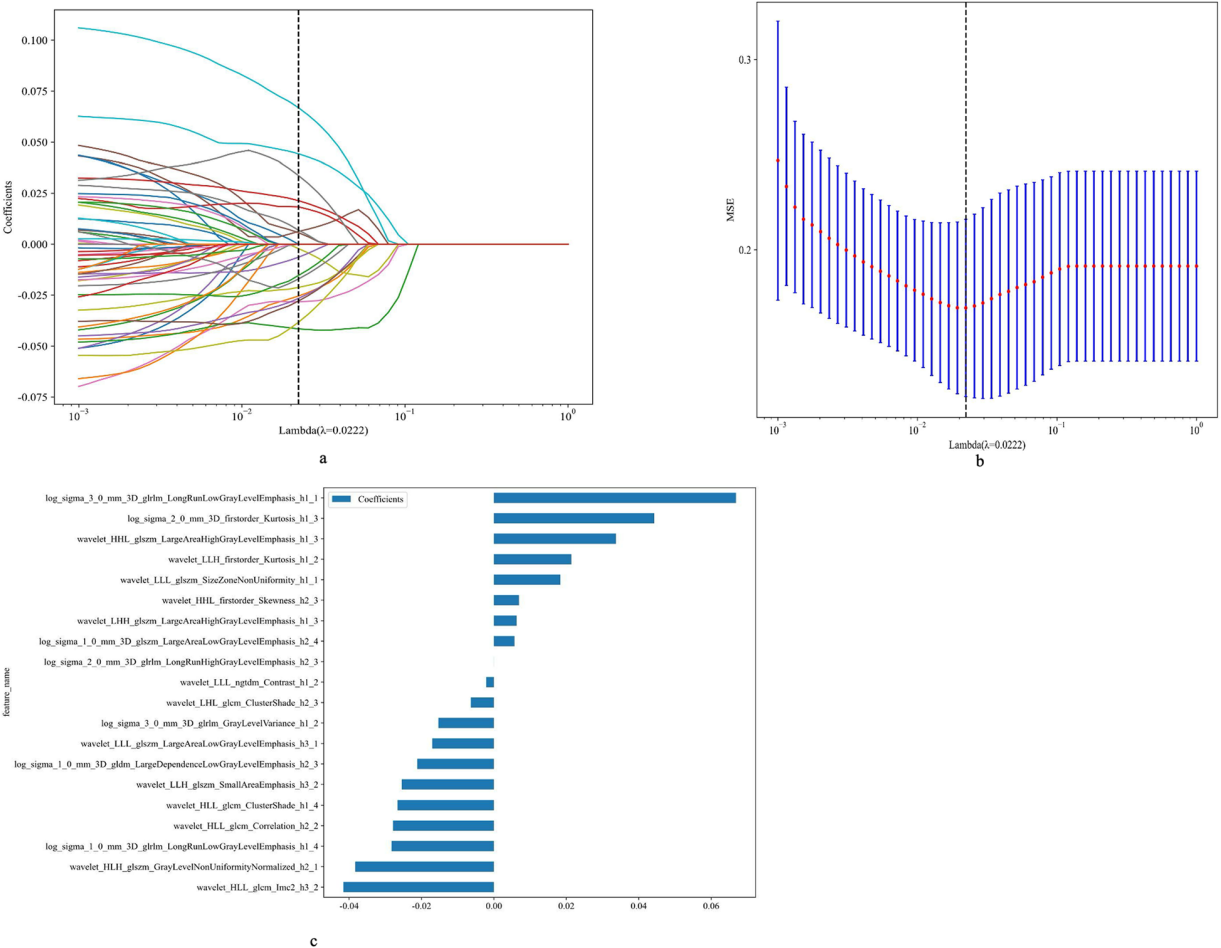
**a** Coefficients of ten-fold cross validation; **b** MSE of ten-fold cross validation; **c** The histogram of the Rad-score based on the selected features. MSE: Mean Standard Error

In our study, we used the Lasso method for feature selection, in particular to identify the non-zero coefficients of the Rad-score using the LASSO logistic regression model. Figure 4 illustrates these coefficients and the Mean Standard Error (MSE) was obtained by a ten-fold cross-validation procedure.

### ITH score

**Fig. 5:**
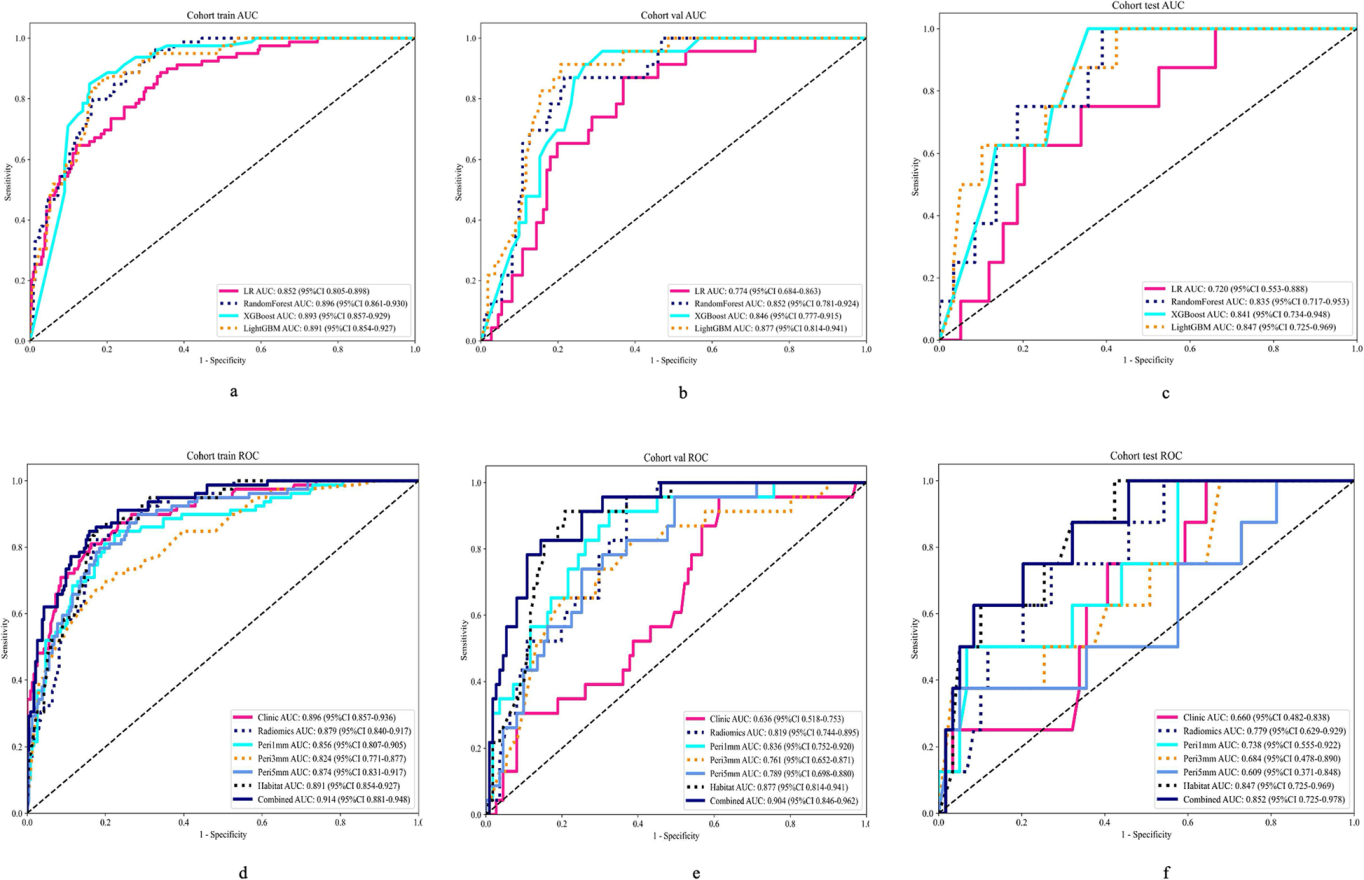
Grading prediction results of ccRCC. ROC Curves of Different Models in each cohort (**a**-**c**). Different signatures'AUROC on all cohort(d-f). LR: Logistic Regression; AUC: area under the curve; Peri: Peritumoral: ROC: receiver operating characteristic

The LightGBM model exhibits the highest area under the curve (AUC) values in all datasets (Fig. 5a-c), specifically, 0.891 for the training set, 0.877 for the validation set, and 0.847 for the test set (Table 2). These results show that the algorithm has excellent discriminative ability in recognizing different categories. In particular, LightGBM's strong AUC performance on both the validation and test sets implies that it has a stable generalization ability. Overall, LightGBM outperforms other algorithms in terms of AUC on all datasets, which confirms its superiority in prediction accuracy. This suggests that LightGBM is able to efficiently recognize patterns in data and provide accurate predictions on both training and new data, making it the best model for this classification task.

**Table 2:** Model performance of different machine learning algorithms in each cohort

model_name	Cohort	Accuracy	AUC	95% CI	Sensitivity	Specificity	PPV	NPV
LR	Training	0. 740	0. 852	0. 805 - 0. 898	0. 772	0. 730	0.492	0. 904
LR	Validation	0. 724	0. 774	0. 684 - 0. 863	0. 652	0. 739	0.341	0. 911
LR	Testing	0. 642	0. 720	0. 553 - 0. 888	0. 750	0. 627	0.214	0. 949
RandomForest	Training	0. 798	0. 896	0. 861 - 0. 930	0. 810	0. 794	0.571	0. 925
RandomForest	Validation	0. 806	0. 852	0. 781 - 0. 924	0. 783	0. 811	0.462	0. 947
RandomForest	Testing	0. 687	0. 835	0. 717 - 0. 953	0. 750	0. 678	0.240	0. 952
XGBoost	Training	0. 840	0. 893	0. 857 - 0. 929	0. 810	0. 850	0.646	0. 930
XGBoost	Validation	0. 776	0. 846	0. 777 - 0. 915	0. 696	0. 793	0.410	0. 926
XGBoost	Testing	0. 731	0. 841	0. 734 - 0. 948	0. 625	0. 746	0.250	0. 936
LightGBM	Training	0. 814	0. 891	0. 854 - 0. 927	0. 861	0. 798	0.591	0. 944
LightGBM	Validation	0. 828	0. 877	0. 814 - 0. 941	0. 826	0. 829	0.500	0. 958
LightGBM	Testing	0. 687	0. 847	0. 725 - 0. 969	0. 875	0. 661	0.259	0. 975

LR：Logistic Regression

### Model evaluation

AUC value analysis: In the peri-tumor region, Peri1 mm had the best performance in training (AUC = 0.856), validation (AUC = 0.836), and test sets (AUC = 0.738) (Fig. 5d-f). As the region expanded to Peri3 mm and Peri5 mm, the AUC values declined, indicating that a broader peritumoral range led to reduced prediction efficiency. The Habitat signature beat the radiomics signature in all datasets (training: AUC = 0.891; validation: AUC = 0.877; test: AUC = 0.847) (Table 3), suggesting detailed subregion characterization is better than treating the tumor as one entity. The DeLong test (Supplementary Fig. 2) compared feature improvement significance in each dataset. The results show that the comprehensive model with Habitat, Peri1 mm, and key clinical features usually performs better than individual models in most cases.

**Table 3:** Prediction performance of Intratumor Heterogeneity Region based rad signatures

Signature	Cohort	Accuracy	AUC	95% CI	Sensitivity	Specificity	PPV	NPV
Clinic	Training	0. 830	0. 896	0. 8567 - 0. 9362	0. 810	0. 837	0. 627	0. 929
Radiomics	Training	0. 788	0. 879	0. 8405 - 0. 9171	0. 861	0. 764	0. 553	0. 942
Peri1mm	Training	0. 798	0. 856	0. 8071 - 0. 9054	0. 785	0. 803	0. 574	0. 917
Peri3mm	Training	0. 718	0. 824	0. 7713 - 0. 8771	0. 734	0. 712	0. 464	0. 888
Peri5mm	Training	0. 811	0. 874	0. 8311 - 0. 9170	0. 722	0. 841	0. 606	0. 899
Habitat	Training	0. 814	0. 891	0. 8539 - 0. 9274	0. 861	0. 798	0. 591	0. 944
Combined	Training	0. 846	0. 914	0. 8812 - 0. 9477	0. 658	0. 910	0. 712	0. 887
Clinic	Validation	0. 560	0. 636	0. 5184 - 0. 7534	0. 565	0. 559	0. 210	0. 861
Radiomics	Validation	0. 716	0. 819	0. 7436 - 0. 8952	0. 652	0. 730	0. 333	0. 910
Peri1mm	Validation	0. 761	0. 836	0. 7522 - 0. 9195	0. 739	0. 766	0. 395	0. 934
Peri3mm	Validation	0. 709	0. 761	0. 6523 - 0. 8706	0. 739	0. 703	0. 340	0. 929
Peri5mm	Validation	0. 754	0. 789	0. 6981 - 0. 8804	0. 565	0. 793	0. 361	0. 898
Habitat	Validation	0. 828	0. 877	0. 8141 - 0. 9407	0. 826	0. 829	0. 500	0. 958
Combined	Validation	0. 858	0. 904	0. 8455 - 0. 9618	0. 652	0. 901	0. 577	0. 926
Clinic	Testing	0. 597	0. 660	0. 4816 - 0. 8384	0. 625	0. 593	0. 172	0. 921
Radiomics	Testing	0. 612	0. 779	0. 6286 - 0. 9286	0. 750	0. 593	0. 200	0. 946
Peri1mm	Testing	0. 582	0. 738	0. 5550 - 0. 9217	0. 750	0. 559	0. 187	0. 943
Peri3mm	Testing	0. 522	0. 684	0. 4783 - 0. 8903	0. 625	0. 508	0. 147	0. 909
Peri5mm	Testing	0. 612	0. 609	0. 3707 - 0. 8475	0. 500	0. 627	0. 154	0. 902
Habitat	Testing	0. 687	0. 847	0. 7255 - 0. 9694	0. 875	0. 661	0. 259	0. 975
Combined	Testing	0. 881	0. 852	0. 7249 - 0. 9785	0. 000	1. 000	0. 000	0. 881

Peri：Peritumoral

**Fig. 6:**
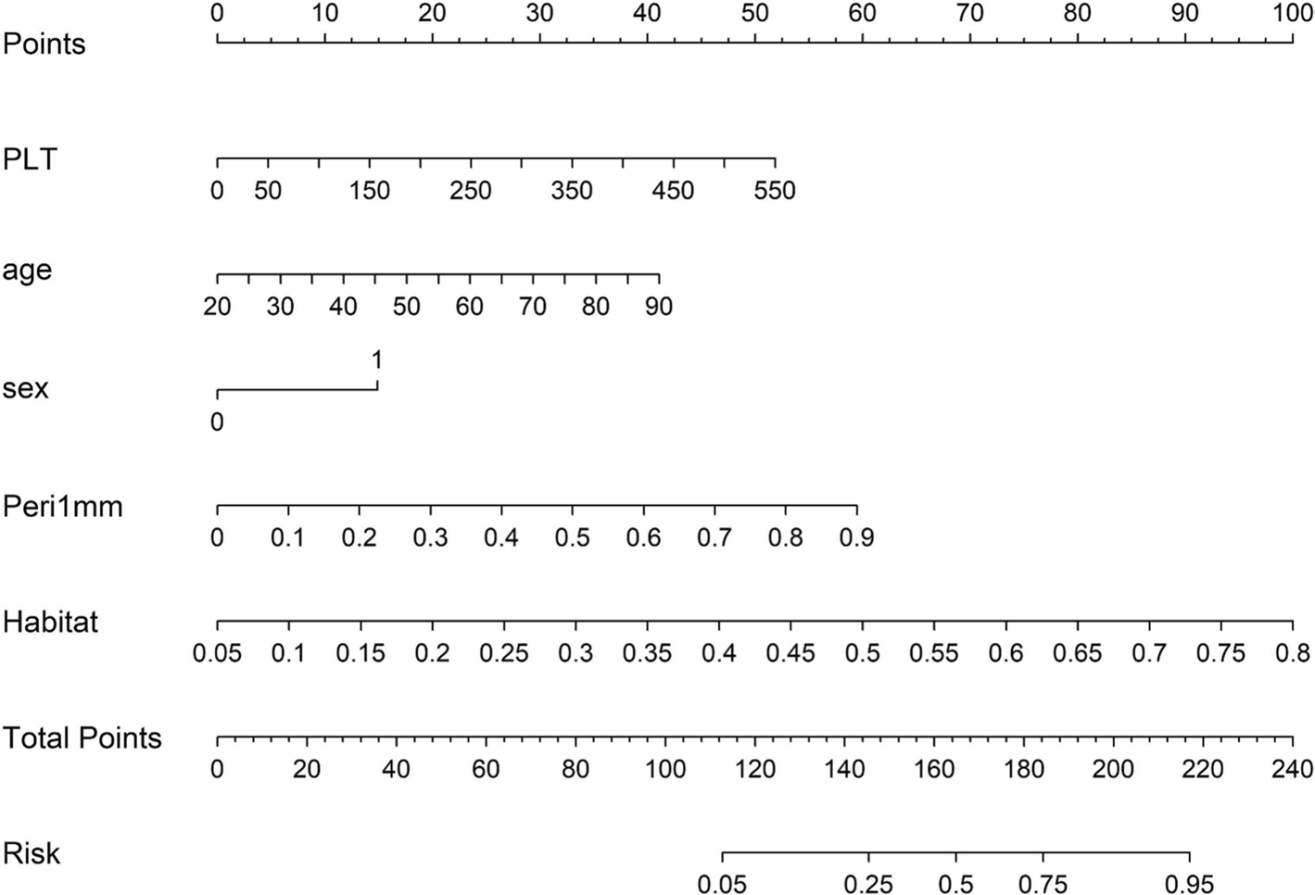
Shows the nomogram for clinical use

Use calibration curves (Supplementary Fig. 3) and decision curves (Supplementary Fig. 4) to assess the model's predictive capabilities. The Hosmer–Lemeshow (HL) test compares predicted probabilities with actual outcomes to measure calibration performance; a lower HL value means better calibration. The nomogram (Fig. 6) was well-calibrated, with HL values of 0.920 in the training dataset, 0.824 in the validation dataset, and 0.812 in the test dataset. The decision curve analysis shows our comprehensive model has a significant net benefit advantage in predicted probabilities. These results highlight the model's excellent predictive accuracy and reliability. In summary, integrating detailed subregional features with optimal peritumoral configurations and clinical characteristics can significantly enhance the precision of predictions, highlighting the advantage of a comprehensive fusion approach in robust outcome prediction.

## Discussion

In the present investigation, we amalgamated radiomics with habitat imaging methodologies, incorporating the examination of peritumoral areas and clinical characteristics, to develop a suite of predictive models. The findings revealed that the LightGBM model demonstrated the most robust performance, yielding the highest AUC values across all datasets: 0.891 for the training set, 0.877 for the validation set, and 0.847 for the test set. The integrated model achieved an AUC of 0.852 on the test set, which represented a statistically significant enhancement over the individual models. Consequently, the model we have developed facilitates a nuanced analysis of different regions of the tumor, markedly enhancing the precision of grading and prognostic evaluation when compared to radiomics models employed in isolation. This advancement supports the deployment of more precision-based clinical strategies.

In recent years, habitat imaging technology has drawn much attention. It uses differences in histopathology and molecular biology to divide regions with different imaging features into multiple subregions of similar or identical heterogeneity [23, 24]. Analyzing the sub-regions of intratumoral heterogeneity is important for many clinical applications like diagnosis and prognosis. Such analyses usually depend on imaging techniques like CT and MRI [25, 26]. The application of habitat imaging technology in the field of renal cancer has gradually emerged. However, the current research [15, 26] primarily focuses on predicting renal cancer metastasis and patient survival prognosis. Yang et al. [26] predicted the metastatic risk in ccRCC through sub-regional radiomics analysis. The study found that a model combining CT and ultrasound imaging features with clinical variables was better at predicting the metastasis of ccRCC. Shan et al. [15] developed and validated a clinical radiomics model based on intra-tumor habitat imaging to predict the progression-free survival of ccRCC patients before surgery. This model had high predictive accuracy in the tested dataset and might be useful in clinical decision-making. However, there are relatively few studies [27] that use CT images combined with habitat imaging technology for the grading of renal cancer. Alhussaini et al. [27] extracted radiomics features from CT images to distinguish between high- and low-grade ccRCC in the preoperative stage and incorporated tumor subregion heterogeneity analysis. The results support that tumor subregions are important factors to consider in the grading of ccRCC.

Therefore, the focus of our study is to improve the accuracy of preoperative grading prediction for ccRCC by adopting a tumor habitat segmentation approach. Initially, we extracted local features, such as entropy and energy, from individual voxels within CT images. Utilizing the K-means clustering algorithm, we grouped these features into clusters, ranging in number from 3 to 10. The optimal cluster count was identified as 3, based on the CH index, which enhances our ability to delineate the intricate heterogeneity within tumors. Following this, we employed the pyradiomics toolkit to extract a wealth of subregion-specific features, encompassing geometric, intensity, and textural attributes. These features provided insights into the tumor's shape, size, luminosity, and spatial distribution patterns. We then leveraged machine learning techniques, including Logistic Regression, Random Forest, XGBoost, and LightGBM, to build predictive models. Our findings revealed that the subregion features we developed significantly outperformed conventional radiomics features across all evaluated datasets, underscoring the utility of our approach in refining the prediction of ccRCC pathological grading. The previous research [28, 29] on kidney cancer grading mainly utilized radiomics methods. Yi et al. [28] developed a CT-based radiomics and machine learning model to predict the pathological grade of ccRCC, which achieved AUC values of 0.8170 and 0.8017 for the training and validation cohorts, respectively. Wang et al. [29] proposed a model based on CT radiomics features for predicting WHO/ISUP grade of ccRCC, which achieved an AUC of 0.89 in the training set and 0.81 in the independent external validation set. In contrast, our study integrated radiomics techniques to perform subregion analysis of tumor heterogeneity and developed a corresponding model. The results demonstrated that the model achieved AUC scores of 0.891, 0.877 and 0.847 in the training set, validation set, and test set, respectively, confirming the superior predictive performance of the habitat-based model.

Additionally, another significant advantage of this study lies in constructing a comprehensive grading model based on clustering analysis, which combines tumor peripheral region characteristics and significant clinical features. Previous studies [30, 31] have demonstrated that the performance of the fusion prediction model integrating intra-peritumoral features is superior to that of only a single model. Huang et al. [30] utilized a habitat-based radiomics approach to evaluate the immediate efficacy of radiofrequency ablation in patients with colorectal cancer lung metastases, which showed that the predictive model combining intratumoral and peritumoral (5 mm dilation) features demonstrated the highest performance. Shi et al. [31] discovered that when predicting lymph node metastasis status in early-stage cervical cancer, peritumoral radiomics features showed higher predictive performance compared to intratumoral features. These results indicate that the peritumoral region contains tumor-related information, which may be related to the invasive growth pattern of malignant tumors into the surrounding tissue. As a type of malignant tumor, ccRCC also has the characteristic of infiltrating into the peritumoral region. Therefore, we incorporated intra-peritumoral features into our study, and results showed that the peritumoral features can effectively improve the predictive performance of the model.

In addition, we extended the peritumoral region by 1 mm (Peri1mm), 3 mm (Peri3mm), and 5 mm (Peri5mm). The results showed that the Peri1 mm configuration exhibited the best performance in the training set (AUC = 0.856), the validation set (AUC = 0.836), and the test set (AUC = 0.738), which indicate that the information of Peri1mm configuration may contain more useful information to distinguish the degree of tumor invasion. Tang et al. [32] used radiomics to distinguish non-small cell lung cancer. They divided the peritumoral region into an inner ring (0–5 mm) and an outer ring (5–10 mm) to study its impact on classification. They found more features in the inner ring, meaning the area closer to the tumor has more information, which agrees with our study. Braman et al. [33] studied whether imaging features inside and around the tumor could show the biological traits of HER2-positive breast cancer. They divided the peritumoral region (extending 15 mm from the tumor margin) into five rings at 3 mm intervals to evaluate patients'response to targeted therapy. The results showed that features 9–12 mm from the tumor edge were better at distinguishing HER2-enriched tumors, and features in the 0–3 mm peritumoral region were related to the density of tumor-infiltrating lymphocytes. This result is different from our study, showing that the usefulness of peritumoral features depends on the analysis goals and the biological nature of the tumor.

Our current research has several limitations. First, although LightGBM showed strong AUC performance in the validation set, the external validation dataset in this study was only sourced from one hospital, which may have certain limitations. In future research, we will enhance the generalization performance of the model from the following aspects: (1) We plan to expand the sample size and increase the diversity of the data. For example, we will include data from different hospitals, various regions, and even different ethnic groups. (2) Establish a standardized set of protocols for data collection, and at the same time, adopt a complete and refined automatic segmentation method to avoid the interference caused by manual segmentation. Second, the delineation of regions of interest depends on radiologists'expertise, which may bring subjective biases. In the future, using deep learning algorithms for automated segmentation can reduce medical professionals'workload and improve image segmentation precision. Third, multiple machine learning models may create overly complex frameworks that are hard to apply in clinical practice. To promote the model's wide application, simpler and more interpretable models should be preferred when possible. Finally, we did not incorporate molecular markers into the model. We will regard this issue as a key direction for future research, striving to broaden and deepen the scope of our studies. In our follow-up work, we aim to deliver more comprehensive and profound results to advance this field.

In conclusion, the radiomics grading prediction model for ccRCC, which integrates habitat imaging with peritumoral features, outperforms conventional radiomics approaches. This suggests that subregion analysis effectively mitigates the impact of tumor heterogeneity, enabling precise preoperative tumor grading. Such precision aids clinicians in more accurately evaluating patient conditions, devising tailored treatment strategies, and ultimately enhancing patient outcomes. In the future, we plan to expand the sample size and enhance multicenter studies to improve the robustness of the model. Additionally, we will establish a dynamic feedback mechanism that enables real-time comparison with pathological results, allowing continuous optimization of the model in clinical settings. Within clinical workflows, we aim to integrate the model into radiology reporting systems. This integration will enable direct output of grading probabilities in imaging reports, thereby assisting diagnostic decision-making.

## Supplementary Information

Supplementary Material 1.

## Data Availability

Data is provided within the manuscript or supplementary information files.

## Abbreviations

WHO/ISUP: The World Health Organization/International Society of Urological Pathology
ccRCC: Clear cell renal cell carcinoma
CT: Computed tomography
ROI: Regions of interest
LASSO: Least Absolute Shrinkage and Selection Operator
ITH: Intratumoral heterogeneity
ROC: Receiver operating characteristic
AUC: Area under the curve
RCC: Renal cell carcinoma
MRI: Magnetic resonance imaging
ICC: Intraclass Correlation Coefficient
