# Supplementary material for: Preoperative prediction of WHO/ISUP grade of ccRCC using intratumoral and peritumoral habitat imaging: multicenter study
Source: Cancer Imaging. 2025 May 3;25:59. doi: 10.1186/s40644-025-00875-z (PMC12049773; doi:10.1186/s40644-025-00875-z)

**Supplementary 1A**

Our methodology for delineating tumor habitat regions was multifaceted and involved several complex steps:

1. **Comprehensive Radiomic Feature Extraction:** This process involved extracting detailed local features from each voxel in the dataset using a 5 x 5 x 5 moving window. These features encompass a variety of measurements and attributes, including intensity, texture, and other statistical properties, which are crucial for understanding the intricate details of the dataset. Such detailed insights enable more precise modeling and analysis.

- In this study, 19 radiomic features were extracted from each voxel, offering a multidimensional characterization of each subregion. These features included a range of shape descriptors, textural features, and first-order statistical attributes. The specific features extracted were: firstorder_Entropy, firstorder_MeanAbsoluteDeviation, firstorder_Median, glcm_DifferenceAverage, glcm_DifferenceEntropy, glcm_DifferenceVariance, glcm_Imc1, glcm_Imc2, glcm_InverseVariance, glcm_JointEnergy, glcm_JointEntropy, glcm_SumEntropy, glrlm_LongRunEmphasis, glrlm_RunEntropy, glrlm_RunVariance, glszm_SizeZoneNonUniformityNormalized, glszm_SmallAreaHighGrayLevelEmphasis, ngtdm_Contrast, and ngtdm_Strength.
- **Entropy**: Entropy specifies the uncertainty/randomness in the image values.

$$entropy=-\sum_{i=1}^{N_{g}} p\left( i \right)\log_{2}\left( p\left( i \right)+\epsilon\right)$$

- **Mean Absolute Deviation (MAD)** : MAD is the mean distance of all intensity values from the Mean Value of the image array.

$$MAD=\frac{1}{N_{p}}\sum_{i=1}^{N_{p}} \left| X\left( i \right)-X \right|$$

- **Difference Entropy**: Measures the randomness/variability in neighborhood intensity value differences.

$$difference\_entropy=\sum_{k=0}^{N_{g}-1} p_{x-y}\left( k \right)\log_{2}\left( p_{x-y}\left( k \right)+\epsilon\right)$$

- **Difference Variance**: A measure of heterogeneity, giving higher weights to differing intensity level pairs.

$$difference\_variance=\sum_{k=0}^{N_{g}-1} \left( k-DA \right)^{2}p_{x-y}\left( k \right)$$

- **Joint Energy**: A measure of homogeneous patterns in the image.

$$joint\_energy=\sum_{i=1}^{N_{g}} \sum_{j=1}^{N_{g}} \left( p\left( i,j \right) \right)^{2}$$

- **Joint Entropy**: Measures the randomness/variability in neighborhood intensity values.

$$joint\_entropy=-\sum_{i=1}^{N_{g}} \sum_{j=1}^{N_{g}} p\left( i,j \right)\log_{2}\left( p\left( i,j \right)+\epsilon\right)$$

1. **KMeans Subregion Clustering:** The K-means algorithm was employed to analyze the multidimensional feature space derived from the radiomic features. This method clustered all voxels and their associated characteristics, exploring a variety of cluster centers, ranging from 3 to 10, to categorize distinct habitat regions within the tumor. The efficacy of the clustering was assessed using the Calinski-Harabasz score, which facilitated the selection of the most statistically significant clustering configuration.

- The K-means algorithm functions by partitioning data into K distinct clusters. It iteratively updates the centroids of these clusters to minimize the sum of squares within each cluster. The central component of the K-means algorithm is the objective function, which is optimized to achieve effective clustering.

$$J=\sum_{i=1}^{N} \sum_{k=1}^{K} w_{ik}\times\parallel x_{i}-\mu_{k}\parallel^{2}$$

- - $J$ is the objective function.
  - $N$ is the number of data points.
  - $K$ is the number of clusters.
  - $w_{ik}$ is a binary indicator (1 if data point $i$ is in cluster $k$, 0 otherwise).
  - $x_{i}$ is the ith data point.
  - $\mu_{k}$ is the centroid of cluster $k$.
  - $\parallel x_{i}-\mu_{k}\parallel^{2}$ is the squared Euclidean distance between data point $i$ and centroid $k$.

**Habitat Region Synthesis:** Following the clustering analysis, subregions with identical cluster IDs were amalgamated. This synthesis resulted in the formation of comprehensive habitat regions, each representing a unique microenvironmental characteristic within the tumor.

**Supplementary Table** :**Patient Clinical Data**

| Feature_name | Training set  (n=312) | Validation set  (n=134) | External test set(n=67) | pvalue |
| --- | --- | --- | --- | --- |
| Sex |  |  |  | 0.637 |
| female | 109(34.94) | 43(32.09) | 22(32.84) |  |
| male | 203(65.06) | 91(67.91) | 45(67.16) |  |
| Age（mean±SD） | 58.13±12.32 | 58.34±12.80 | 58.03±10.93 | 0.83 |
| Hematuria |  |  |  | 0.755 |
| absent | 284(91.03) | 120(89.55) | 61(91.04) |  |
| present | 28(8.97) | 14(10.45) | 6(8.96) |  |
| Abdominalpain |  |  |  | 0.203 |
| absent | 247(79.17) | 98(73.13) | 64(95.52) |  |
| present | 65(20.83) | 36(26.87) | 3(4.48) |  |
| Abdominalmass |  |  |  | 0.326 |
| absent | 307(98.40) | 134(100.00) | 67(100.00) |  |
| present | 5(1.60) | 0 | 0 |  |
| Tumorhistory |  |  |  | 0.308 |
| absent | 291(93.27) | 129(96.27) | 52(77.61) |  |
| present | 21(6.73) | 5(3.73) | 15(22.39) |  |
| Location |  |  |  | 0.869 |
| left | 158(50.64) | 66(49.25) | 34(50.75) |  |
| right | 154(49.36) | 68(50.75) | 33(49.25) |  |
| eGFR(ml/(min·1.73m2)) | 82.29±16.27 | 80.73±13.77 | 83.97±26.45 | 0.532 |
| BUN(mmol/L) | 5.79±1.73 | 5.90±1.58 | 6.07±3.29 | 0.361 |
| Cr(umol/L) | 73.54±39.93 | 71.51±20.11 | 110.83±136.10 | 0.891 |
| CHO(mmol/L) | 4.44±0.77 | 4.58±0.96 | 4.52±0.38 | 0.163 |
| ALB(g/L) | 39.73±4.77 | 39.94±4.12 | 8.05±3.26 | 0.708 |
| Hb(g/L) | 129.51±17.69 | 130.76±18.73 | 131.61±19.69 | 0.352 |
| PLT(g/L) | 205.55±72.16 | 213.04±64.44 | 225.96±68.82 | 0.154 |
| NE(10×10^9) | 3.71±1.63 | 4.19±4.87 | 58.77±29.61 | 0.545 |
| LYM(10×10^9) | 1.82±1.25 | 1.97±2.61 | 16.45±12.44 | 0.846 |
| MO(10×10^9) | 0.50±0.19 | 0.56±0.60 | 5.21±2.91 | 0.556 |
| R(mm) | 52.11±25.91 | 49.47±22.47 | 50.20±24.94 | 0.572 |
| E |  |  |  | 0.749 |
| exophytic/endophytic≥50％ | 144(46.15) | 67(50.00) | 27(40.30) |  |
| exophytic/endophytic＜50％ | 154(49.36) | 61(45.52) | 32(47.76) |  |
| endophytic | 14(4.49) | 6(4.48) | 8(11.94) |  |
| N |  |  |  | 0.112 |
| N≥7mm | 50(16.03) | 32(23.88) | 9(13.43) |  |
| 4＜N＜7mm | 33(10.58) | 10(7.46) | 7(10.45) |  |
| N≤4mm | 229(73.40) | 92(68.66) | 51(76.12) |  |
| A |  |  |  | 0.492 |
| ventral | 115(36.86) | 56(41.79) | 20(29.85) |  |
| dorsal | 92(29.49) | 40(29.85) | 27(40.30) |  |
| uncertain location | 105(33.65) | 38(28.36) | 20(29.85) |  |
| L |  |  |  | 0.452 |
| completely above the upper pole line or below the lower pole line | 92(29.49) | 47(35.07) | 19(28.36) |  |
| crosses the polar line but does not exceed 50% of the diameter | 92(29.49) | 39(29.10) | 22(32.84) |  |
| crosses more than 50% of the polar line, or the tumor crosses the renal midline, or the tumor is completely between the polar lines. | 128(41.03) | 48(35.82) | 26(38.81) |  |
| H |  |  |  | 0.259 |
| no | 260(83.33) | 118(88.06) | 62(92.54) |  |
| yes | 52(16.67) | 16(11.94) | 5(7.46) |  |
| Renal_score | 8.07±1.87 | 7.80±1.95 | 8.31±1.75 | 0.153 |
| Map1(mm) |  |  |  | 0.466 |
| 0＜10 | 186(59.62) | 80(59.70) | 35(52.24) |  |
| 10-19 | 91(29.17) | 34(25.37) | 22(32.84) |  |
| ≥20 | 35(11.22) | 20(14.93) | 10(14.93) |  |
| Map2 |  |  |  | 0.665 |
| no involvement (no changes in perirenal fat) | 108(34.62) | 47(35.07) | 26(38.81) |  |
| **moderate involvement:**Increased density of perirenal fat with fine stranding, but no thickened inflammatory strands | 150(48.07) | 69(51.49) | 31(46.27) |  |
| **severe involvement: Increaseddensity of perirenal fat, presence of disorganized coarse**  **striations, and thick inflammatory bands** | 54(17.31) | 18(13.43) | 10(14.93) |  |
| Map_score | 1.98±1.44 | 1.97±1.47 | 1.96±1.52 | 0.835 |
| Cystic |  |  |  | 0.447 |
| absent | 60(19.23) | 21(15.67) | 14(20.90) |  |
| present | 252(80.77) | 113(84.33) | 53(79.10) |  |
| Capsule |  |  |  | 0.817 |
| absent | 217(69.55) | 91(67.91) | 53(79.10) |  |
| present | 95(30.45) | 43(32.09) | 14(20.90) |  |
| MVD |  |  |  | 0.927 |
| absent | 111(35.58) | 49(36.57) | 11(16.42) |  |
| present | 201(64.42) | 85(63.43) | 56(83.58) |  |
| Calcification |  |  |  | 0.472 |
| absent | 255(81.73) | 114(85.07) | 63(94.03) |  |
| present | 57(18.27) | 20(14.93) | 4(5.97) |  |
| Invasion |  |  |  | 0.789 |
| no | 263(84.29) | 115(85.82) | 62(92.54) |  |
| yes | 49(15.71) | 19(14.18) | 5(7.46) |  |
| Cavainvolvement |  |  |  | 1.0 |
| absent | 292(93.59) | 126(94.03) | 63(94.03) |  |
| present | 20(6.41) | 8(5.97) | 4(5.97) |  |
| Lymphnodes |  |  |  | 1.0 |
| absent | 299(95.83) | 128(95.52) | 65(97.01) |  |
| present | 13(4.17) | 6(4.48) | 2(2.99) |  |
| Metastasis |  |  |  | 0.786 |
| absent | 307(98.40) | 133(99.25) | 67(100.00) |  |
| present | 5(1.60) | 1(0.75) | 0 |  |
| Enhancement1 |  |  |  | 0.38 |
| heterogeneous | 284(91.03) | 126(94.03) | 63(94.03) |  |
| homogeneous | 28(8.97) | 8(5.97) | 4(5.97) |  |
| Enhancement2 |  |  |  | 0.467 |
| absent | 101(32.37) | 38(28.36) | 8(11.94) |  |
| present | 211(67.63) | 96(71.64) | 59(88.06) |  |

NOTE:R= maximum tumor diameter E= exophytic/endophytic N=distance between the tumor and the renal sinus and collecting system A= tumor located on the ventral or dorsal side of the kidney L= tumor location along the longitudinal axis of the kidney H= whether the tumor invades the renal pedicle vessels Map1=posterior perirenal fat thickness at the level of the renal vein Map2=perirenal fat involvement MVD=intratumoral blood vessels Enhancement2=fast in and fast out

**Supplementary Fig. 1.** Number and ratio of handcrafted features.


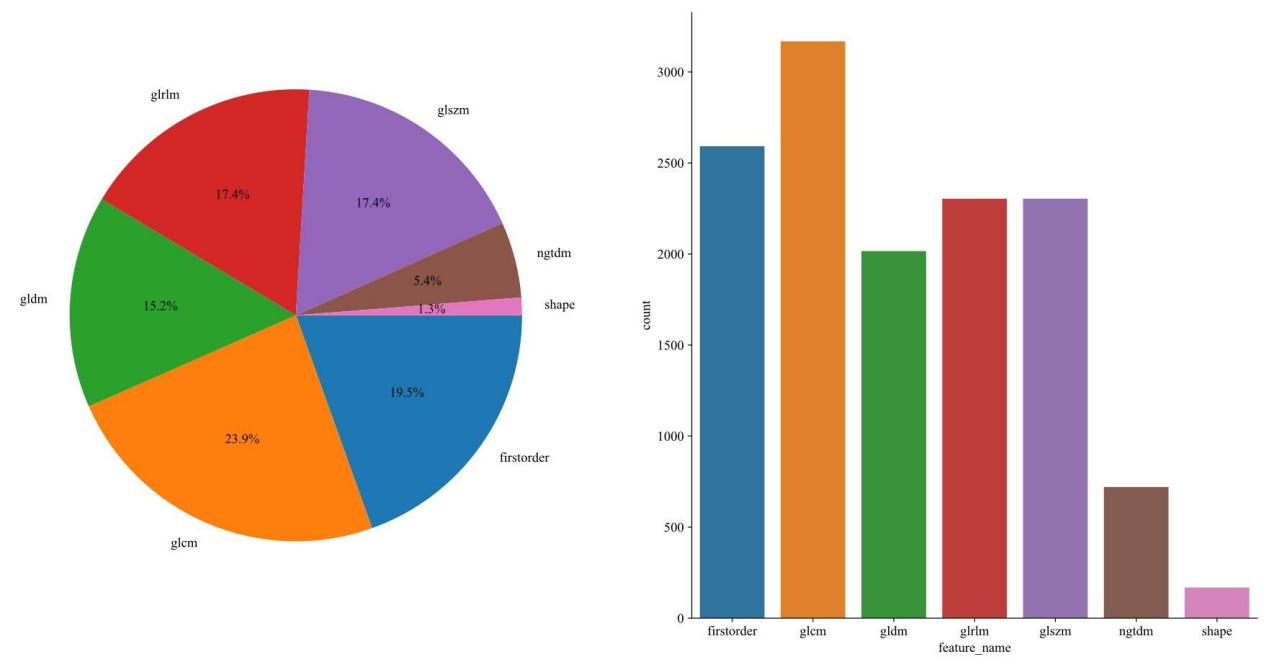


**Supplementary Fig. 2** Delong et. al. of different signature.


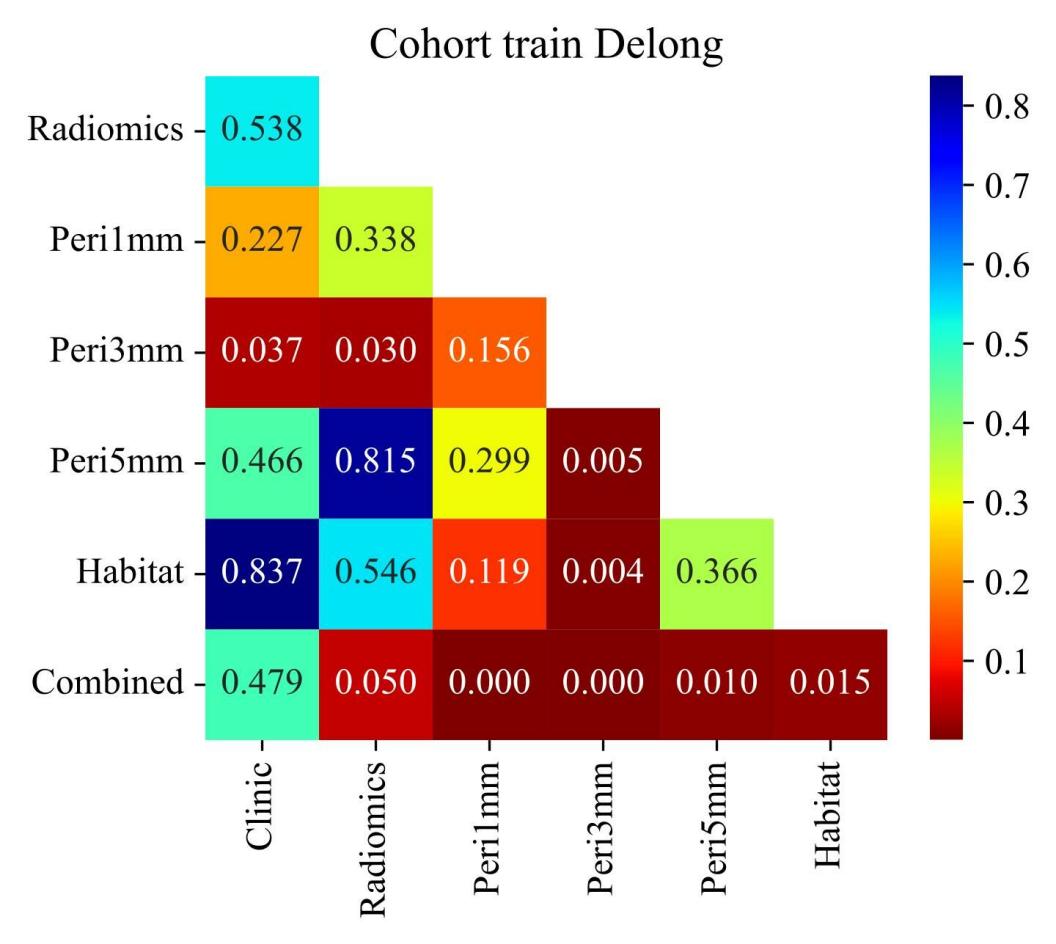


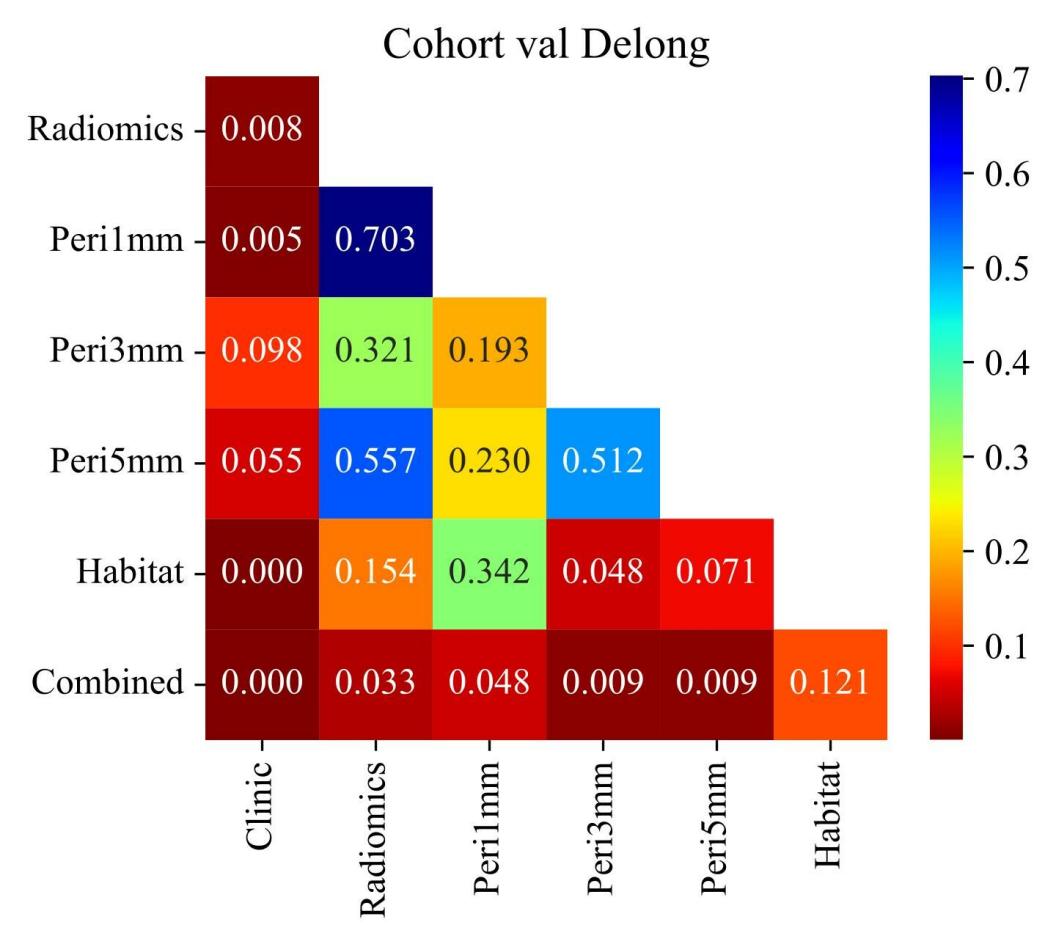


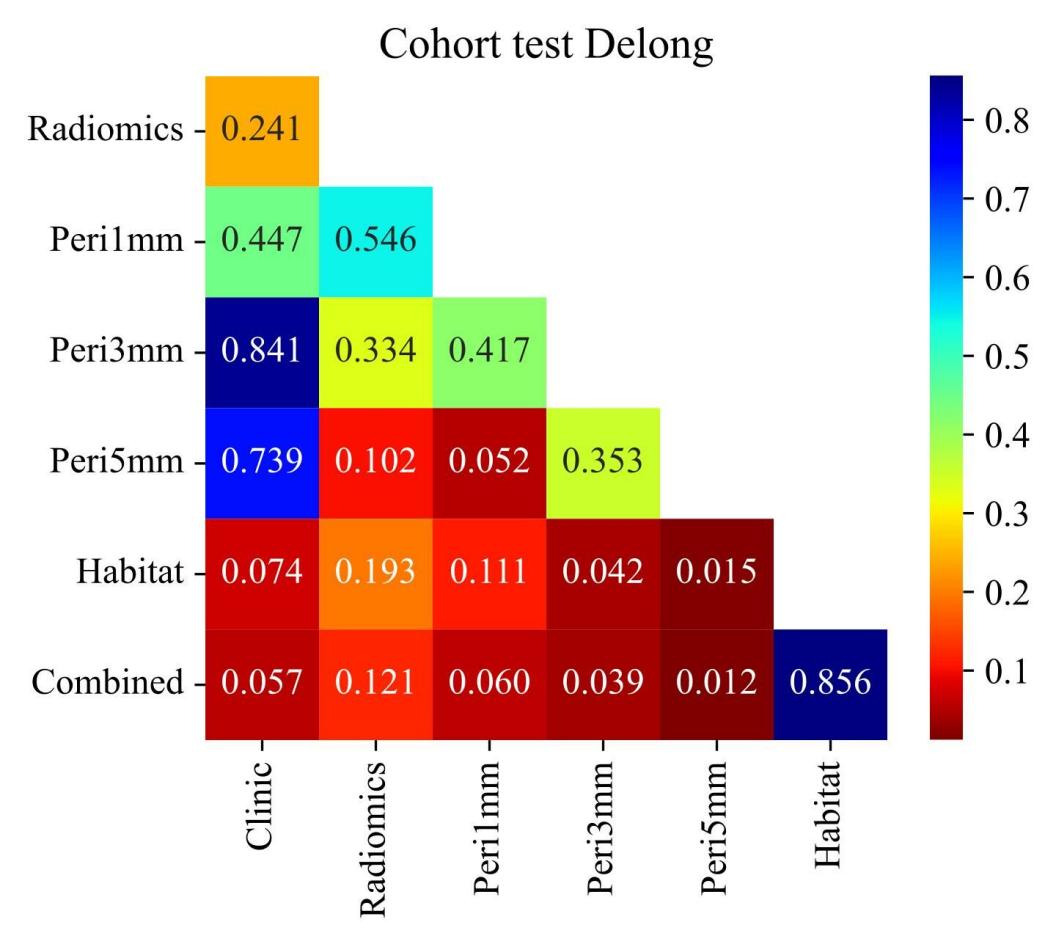


**Supplementary Fig. 3** Different signatures' calibration curve on test cohort.


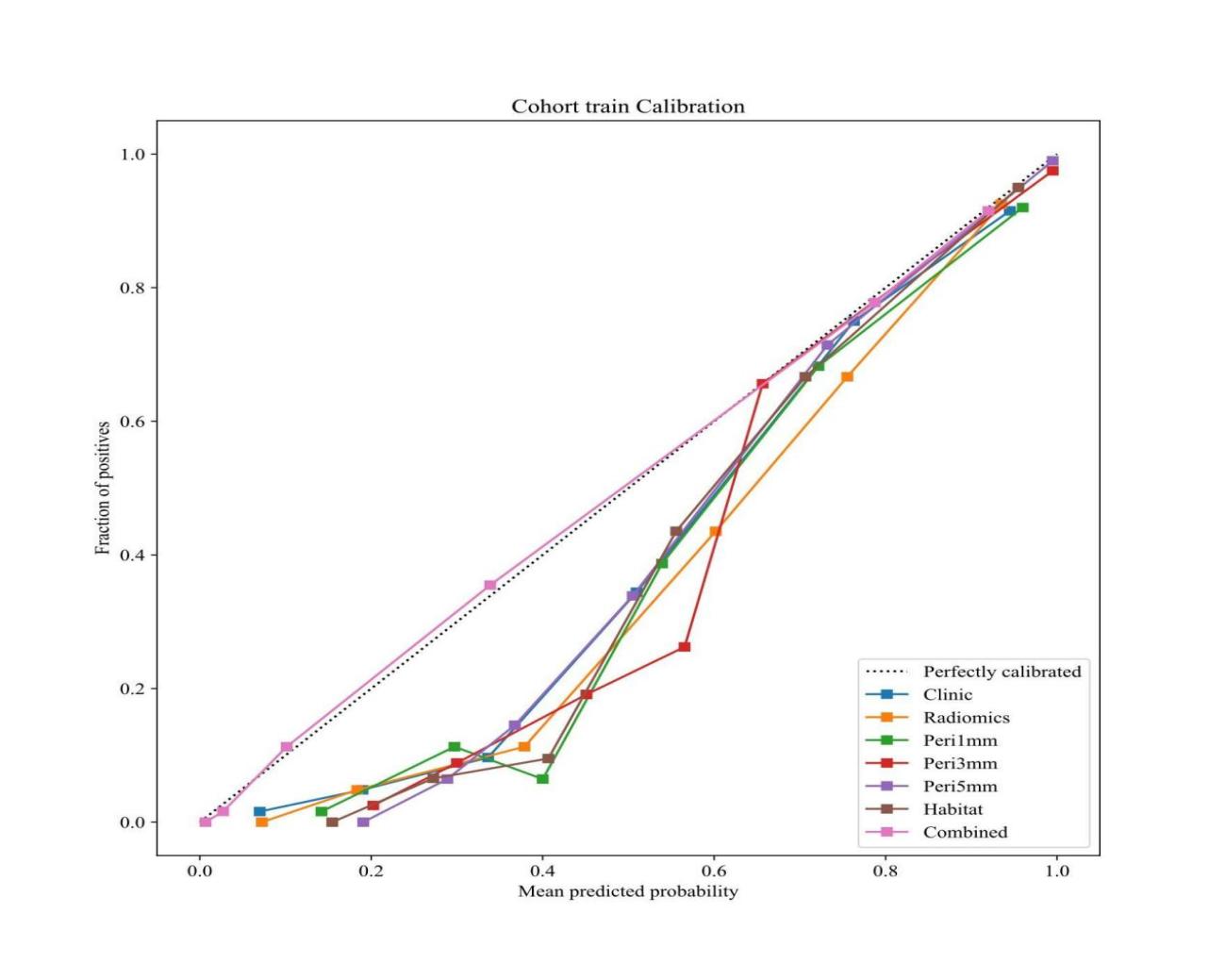


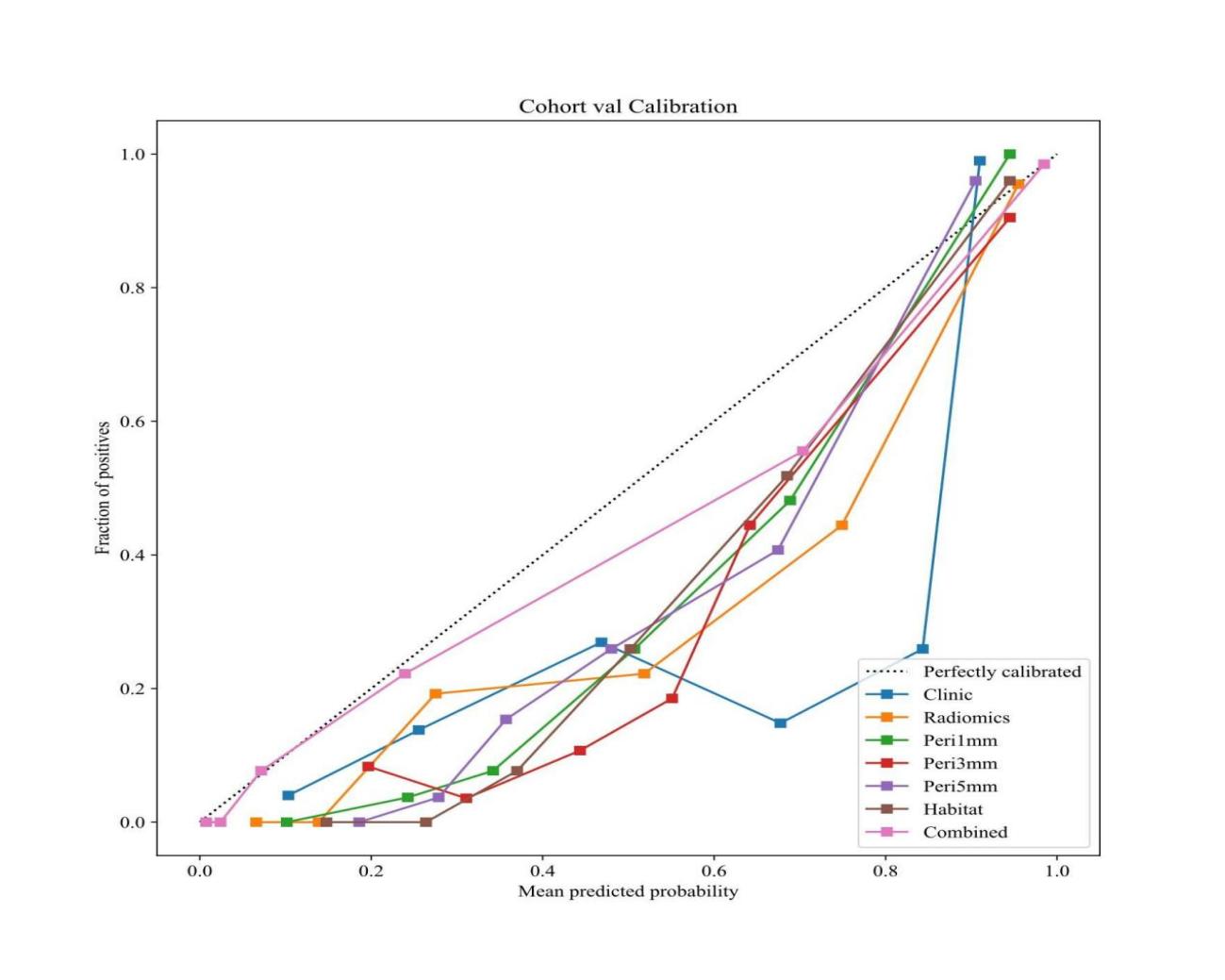


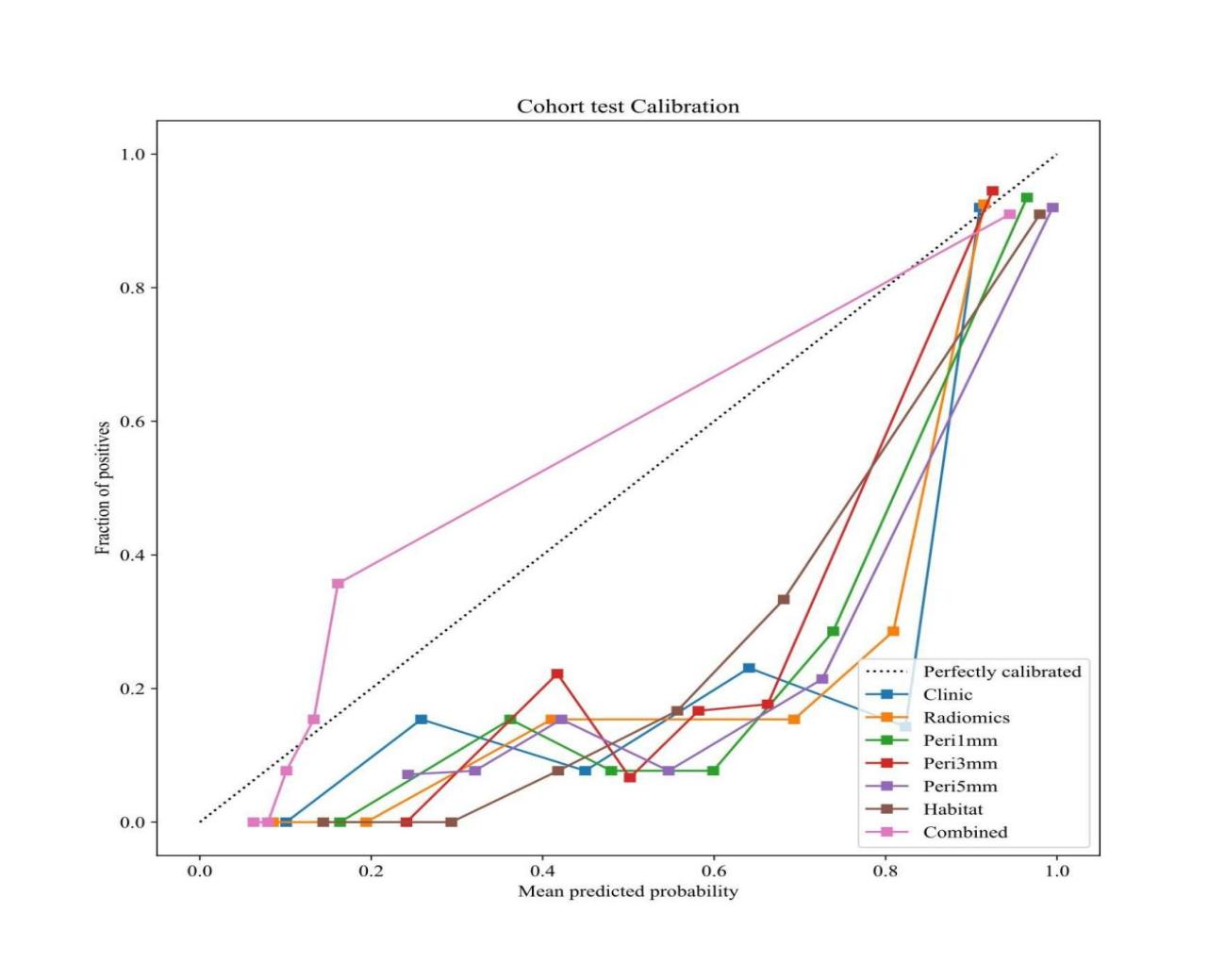


**Supplementary Fig. 4** Different signatures' decision curve on test cohort.


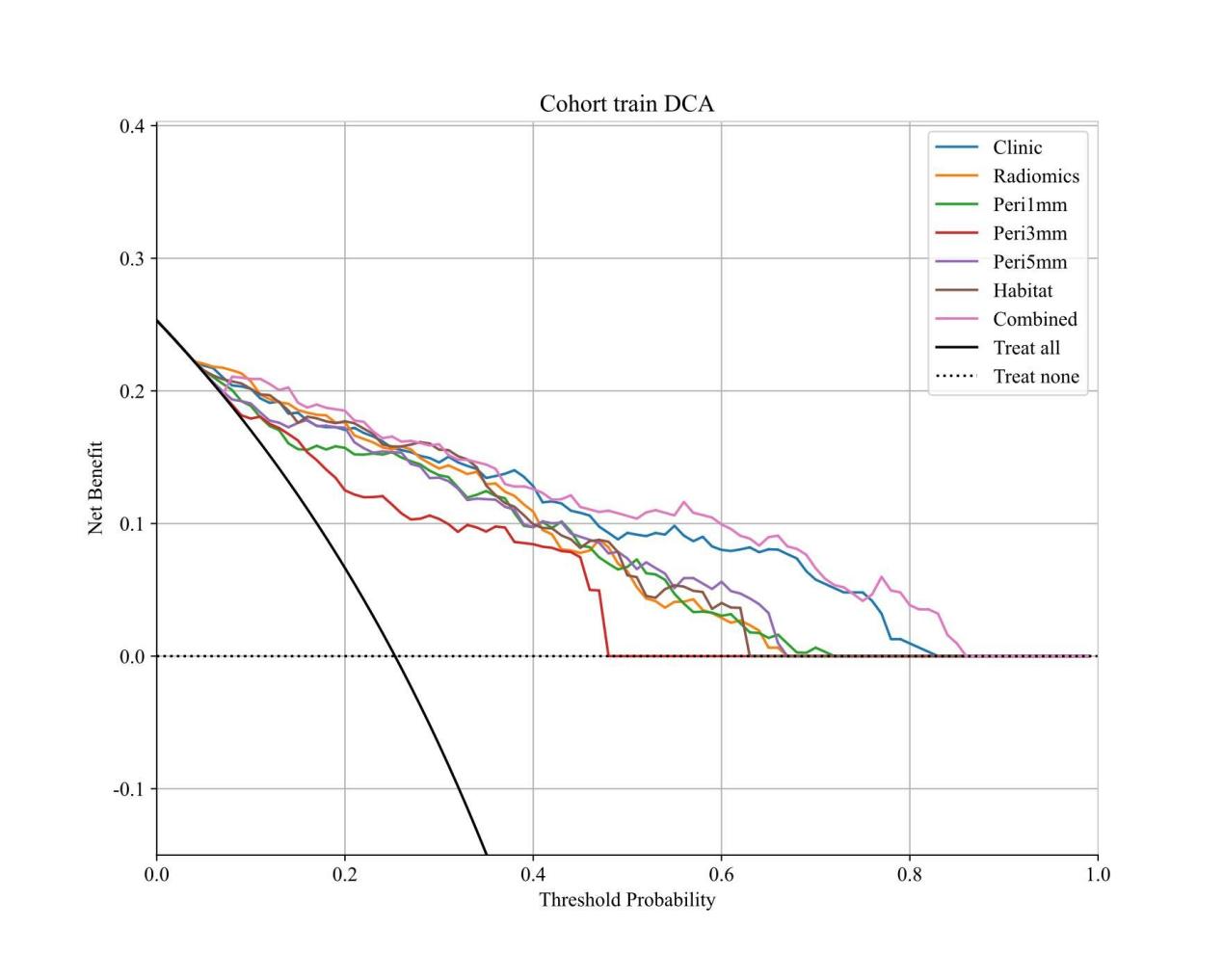


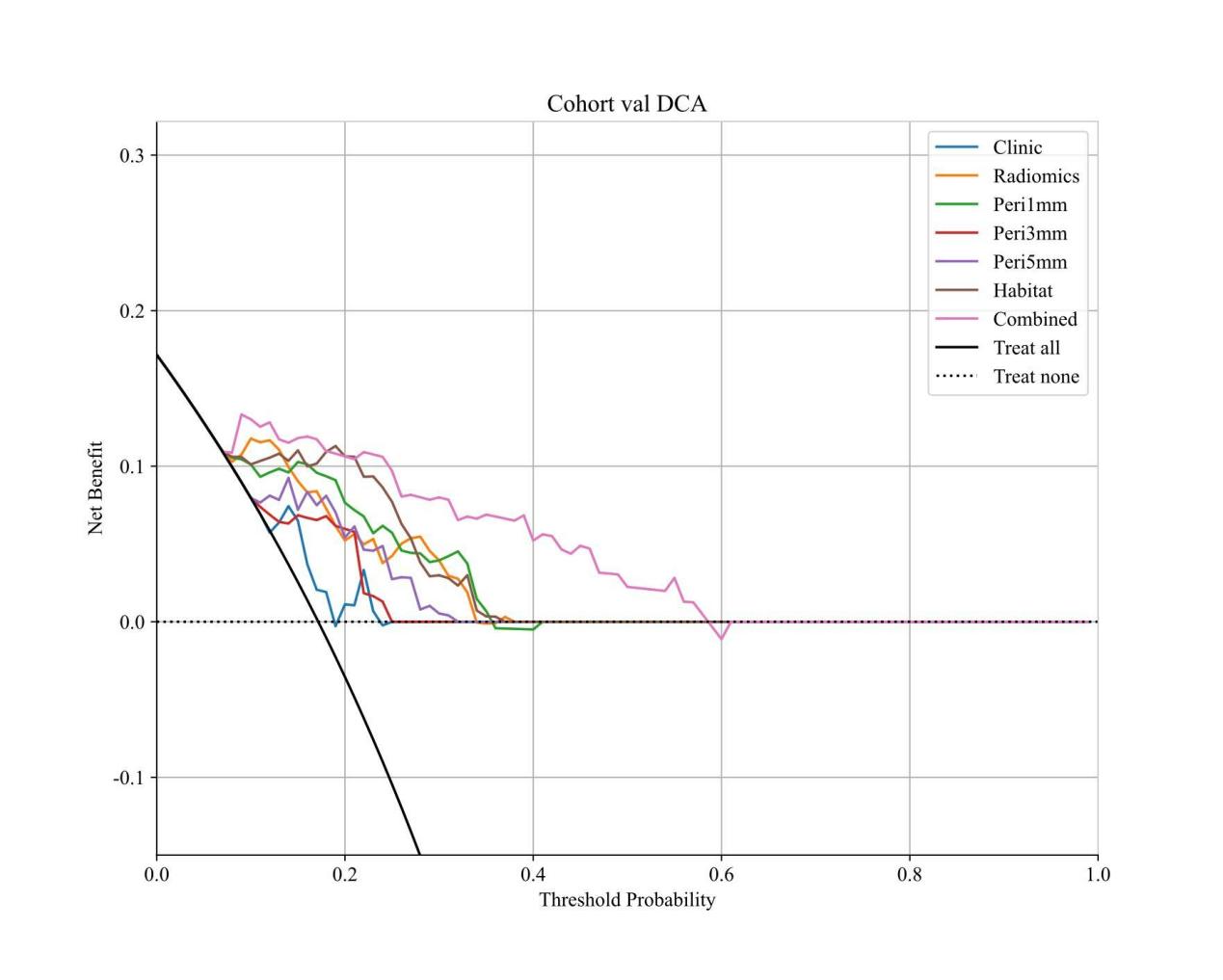


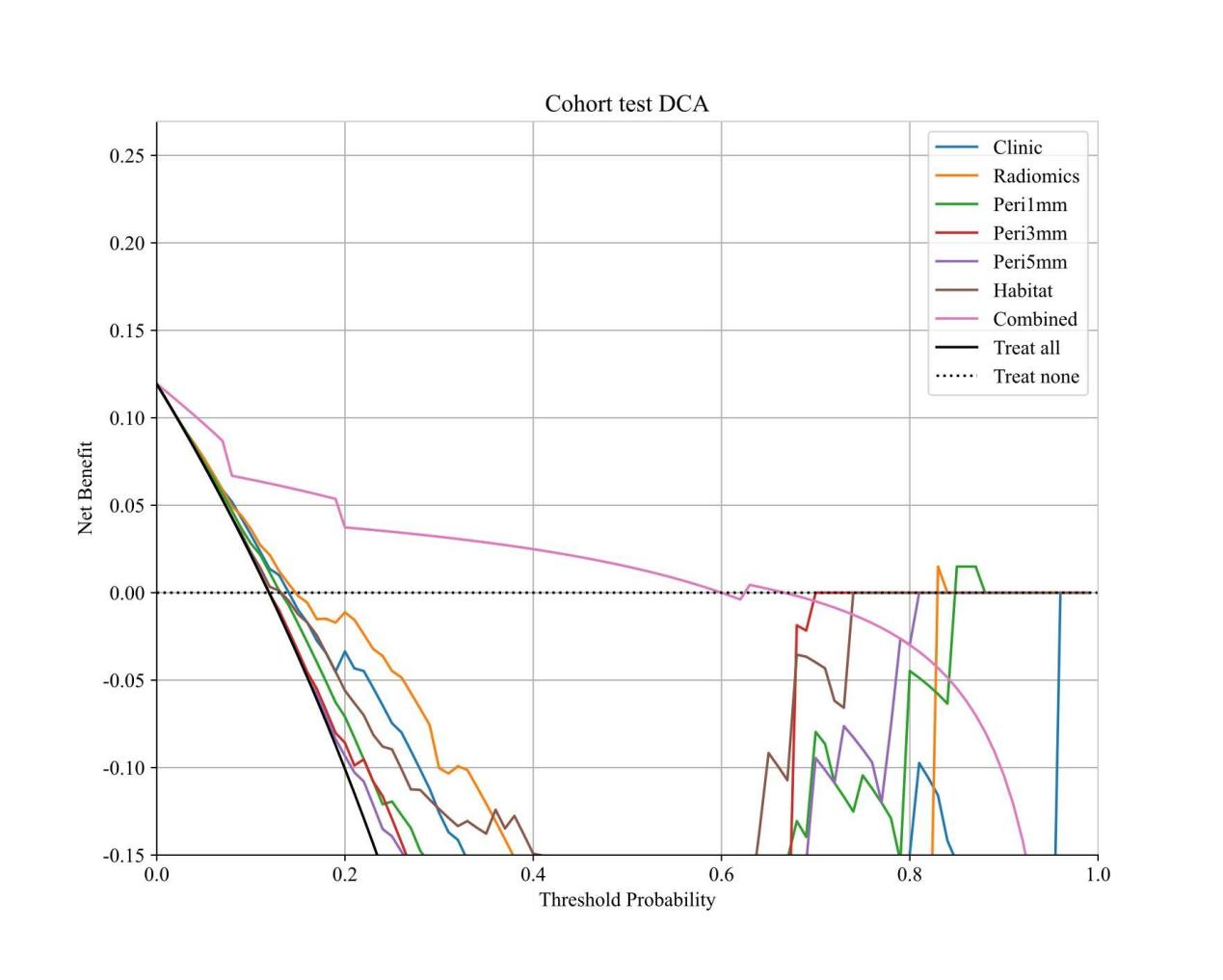

Supplement: Supplementary file 1 — Supplementary Material 1. [file 40644_2025_875_MOESM1_ESM.docx]
